# Long non-coding RNAs in regulation of adipogenesis and adipose tissue function

**DOI:** 10.7554/eLife.59053

**Published:** 2020-07-30

**Authors:** Tiziana Squillaro, Gianfranco Peluso, Umberto Galderisi, Giovanni Di Bernardo

**Affiliations:** 1 Department of Experimental Medicine, Biotechnology, and Molecular Biology Section, University of Campania Luigi VanvitelliNaplesItaly; 2 Institute Bioscience and BioResources, CNRNaplesItaly; Maine Medical Center Research InstituteUnited States; Maine Medical Center Research InstituteUnited States

**Keywords:** lncRNA, adipogenesis, obesity, brown fat, white fat, Human

## Abstract

Complex interaction between genetics, epigenetics, environment, and nutrition affect the physiological activities of adipose tissues and their dysfunctions, which lead to several metabolic diseases including obesity or type 2 diabetes. Here, adipogenesis appears to be a process characterized by an intricate network that involves many transcription factors and long noncoding RNAs (lncRNAs) that regulate gene expression. LncRNAs are being investigated to determine their contribution to adipose tissue development and function. LncRNAs possess multiple cellular functions, and they regulate chromatin remodeling, along with transcriptional and post-transcriptional events; in this way, they affect gene expression. New investigations have demonstrated the pivotal role of these molecules in modulating white and brown/beige adipogenic tissue development and activity. This review aims to provide an update on the role of lncRNAs in adipogenesis and adipose tissue function to promote identification of new drug targets for treating obesity and related metabolic diseases.

## Introduction

Obesity is defined as a chronic, multifactorial condition characterized by excessive adiposity and associated with various chronic diseases. In most cases, it is caused by an unhealthy lifestyle: food and drink intakes that exceed what is needed for metabolic and physical functions, exacerbated by lack of physical activity and sedentary behavior. Genetic and epigenetic factors can also contribute to obesity. Extra energy is stored in extra fat depots. The term *obesity* denotes too much body fat.

Adipose tissue is no longer considered a static tissue that stores excess energy in fat depots: it is considered a real, metabolically-active endocrine organ that is able to produce several hormones and signaling molecules (adipokines) that play a key role in regulating energy homoeostasis, satiety, blood pressure, and angiogenesis. In humans and other mammals, adipocytes originate in stem cells, which are present in mesenchymal stromal cells (MSCs). Human MSCs are heterogeneous and include: i) multipotent stem cells that can differentiate into osteocytes, chondrocytes, and adipocytes; ii) progenitor cells; iii) stromal cells; and iv) fibroblasts. MSCs were first isolated from bone marrow and then from other tissues with a stromal component, including adipose tissue, endometrium, Wharton’s jelly, dental pulp, and the umbilical cord (Squillaro et al., 2016).

Anatomically and physiologically, adipose tissue can be classified into brown adipose tissue (BAT) and white adipose tissue (WAT), with each having different functions. BAT specializes in spreading energy by producing heat. Brown adipocytes present in BAT are characterized by a high number of mitochondria, and they generate heat via the UCP1 protein (uncoupling protein 1) (Iwase et al., 2020). BAT depots in small mammals are located in well-defined anatomical regions and remain metabolically active and stable throughout the animal’s lifetime. In humans, BAT depots are dispersed and will decrease with age (González-Muniesa et al., 2017).

WAT occurs due to a low presence of blood vessels and appears as a yellowish tissue. White adipocytes present a single lipid droplet containing triglycerides and vary in the number of mitochondria. In addition to the brown adipocytes present in BAT, human WAT contains adipocytes that possess the ability to transdifferentiate into ‘brown-like’ cells following thermogenic stimuli or pharmacological treatments. This process is called WAT ‘browning’, and these brown adipocytes are known as ‘inducible beige/brite’ (brown-white). Brite adipocytes express UCP1 and may potentially become heat generators that dissipate energy (Chen et al., 2016; Giralt and Villarroya, 2013).

In terms of body distribution, WAT can be classified into subcutaneous adipose tissue (sWAT)—located under the skin—and visceral adipose tissue (vWAT)—found near the primary organs within the abdominal cavity. Thus, abdominal obesity reflects vWAT growth, which is associated with an increased risk of obesity-related disorders such as insulin resistance, cardiovascular diseases, and hyperlipidemia (Ascaso, 2008).

Several studies have demonstrated that, in addition the well-known high-caloric intake, genetic and epigenetic factors play a role in obesity (Bell, 2017; Tam et al., 2019). Indeed, white and brown adipose tissue biogenesis is regulated by specific gene expression programs, which can vary greatly across individuals. In recent decades, many findings have focused on the role of adipose-specific proteins in the pathophysiology of WAT and BAT. Recently, the role of RNAs—and, in particular, of noncoding RNAs (ncRNAs)—has been investigated to determine their contribution to adipose tissue development and function. Many studies have evaluated the activity of small noncoding RNAs (sncRNAs), such as miRNAs, in BAT and WAT biology. A few investigations have addressed the role of long noncoding RNAs (lncRNAs) in regulation of adipose tissue activities (Sun et al., 2013).

**Figure 1.**
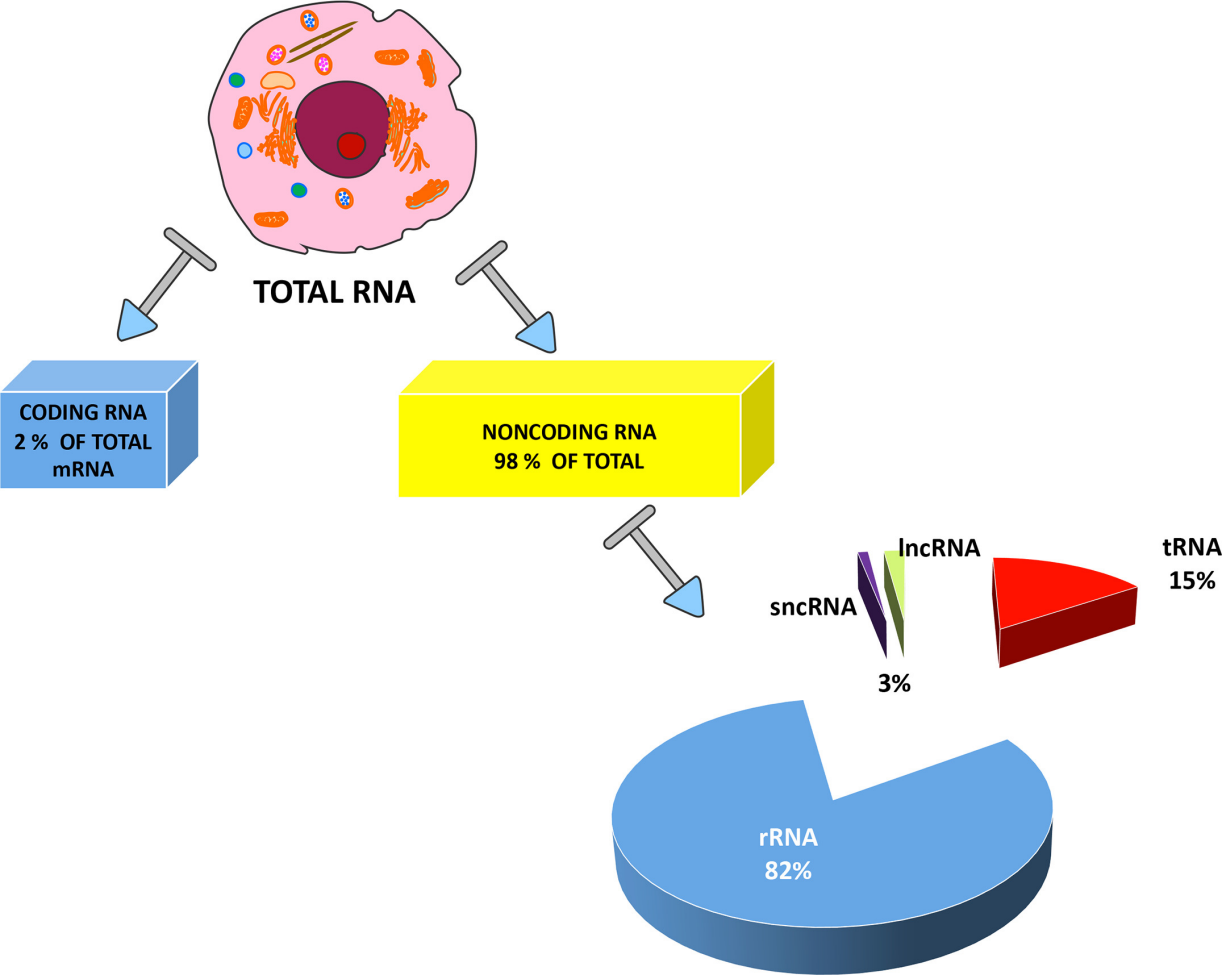
Types of RNAs present in eukaryotic cells. RNAs are primarily divided between coding RNAs and noncoding RNAs. Coding RNAs contain one class of molecules: the messenger RNAs (mRNAs) that undergo the translation process. The other category contains noncoding RNAs, since they are not translated into proteins. Ribosomal RNAs (rRNAs) and transfer RNAs (tRNAs) are the two most abundant classes of noncoding RNAs, but several other RNA types have specific roles in eukaryotic cells. These other RNAs are usually divided into two groups: short noncoding RNAs (sncRNAs), consisting of RNAs with a length of less than 200 nucleotides; and long noncoding RNAs (lncRNAs). The exact percentages of the various ncRNA classes are still under debate and the indicated values are reported in many studies.

Large-scale microarray and comparative genomics analyses suggest that the transcriptome—the collection of all RNA molecules present in one cell—is substantially greater than previously hypothesized (Babak et al., 2005). Only a small fraction of the mammalian genome is transcribed into mRNAs (2%), while most of what was formerly known as ‘junk DNA’ is transcribed into various noncoding RNAs. These do not present open reading frames (ORFs) and hence do not encode proteins (Figure 1).

In general, ncRNAs are grouped into two categories, based on transcript length. RNAs shorter than 200 nucleotides are identified as sncRNAs, such as snRNAs, snoRNAs, miRNAs, and piRNAs. LncRNAs represent the other category and include RNA molecules with an average length longer than 200 nucleotides (West and Lagos, 2019). These RNAs include rRNA (ribosomal RNA), mRNA-like intergenic transcripts (lincRNAs), and natural antisense transcripts (NAT) that are spontaneously transcribed from the sense strands of coding genes (Katayama et al., 2005).

The functions of lncRNAs are largely dependent on their subcellular localization. LncRNAs are primarily localized in the nucleus and are involved in the regulation of gene expression (Rinn and Chang, 2012; Wilusz et al., 2009).

In general, nuclear-retained lncRNAs modulate gene expression by acting either close (‘in cis’) or far (‘in trans’) from transcription sites. Several clues suggest that lncRNAs are transcribed from intergenic regions by RNA polymerase II. Indeed, lncRNAs show mRNA features: they are 5’-end capped, spliced, and polyadenylated (Rege et al., 2015).

Are lncRNAs really noncoding molecules? Very recent studies have indicated the existence of small polypeptides (less than 100 amino acids in length) that, in some cases, can be encoded by lncRNAs (Huang et al., 2017). These polypeptides interact with RNAs and play a key role in some cancers, which can direct research on novel drug targets and biomarkers (Zhu et al., 2018; Wang et al., 2019b).

Even though the role of many lncRNAs is uncertain, an increasing amount of research is trying to characterize their potential function as transcriptional regulators involved in human diseases, development, and differentiation (Yao et al., 2019; Bhat et al., 2016; Babak et al., 2005; Loewer et al., 2010).

Cytoplasmic lncRNAs are less investigated, but new studies have shown that they can form complexes with other biomolecules with structural and regulatory functions. In particular, cytoplasmic lncRNAs can modulate mRNA stability, translation, and protein phosphorylation. Some cytoplasmic lncRNAs hybridize with miRNAs and avoid their binding with mRNA targets (lncRNA sponge activity) (Rashid et al., 2016).

**Figure 2.**
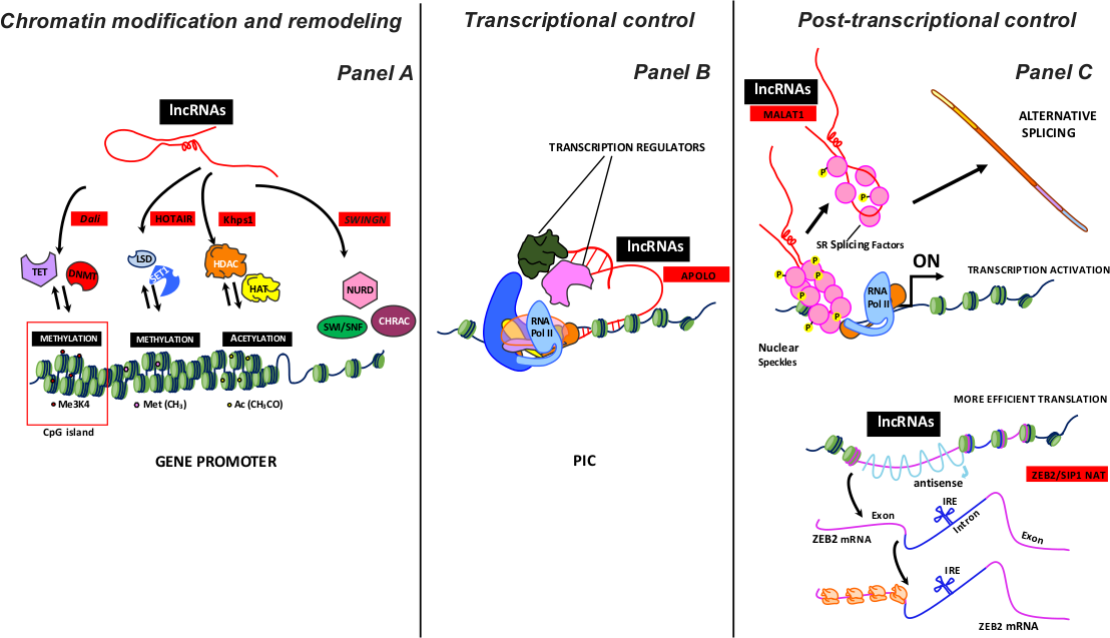
Some examples of lncRNA activity as a regulator of gene expression. LncRNAs, working on proximal loci or at a great distance, can inhibit or activate the expression gene at various levels: i) chromatin modification and remodeling; ii) transcriptional control, and iii) post-transcriptional control. Panel A summarizes the different chromatin remodeling mechanisms. From left to right: nucleotide covalent modifiers (methylation) at CpG island (TET, DNMT enzymes); histone covalent modifiers, which regulate amino acid methylation (LSD, SET1 enzymes) and acetylation (HADC and HAT enzymes); ATP-dependent chromatin remodeling complexes (SWI/SNF, NURD, CHRAC). The lncRNAs acting with different enzymatic complexes are depicted with red squares. Panel B shows lncRNAs involved in the early transcription stages. LncRNAs acquiring a specific R-loops structure interact with PIC (Preinitiation Complex) to modulate transcription by recruiting regulator factors. The lncRNA acting with different transcription regulators is depicted with a red square. Panel C shows some post-transcription activities of lncRNAs. Top: MALAT1 localizes to interchromatin granule clusters (nuclear speckles) and regulates alternative splicing by modulating the distribution and the levels of active SR splicing factors. Bottom: ZEB2/SIP1 NAT binds and masks specific splicing sites, causing intron retention. The retained intron contains an IRES site (internal ribosome entry site) that induces a more efficient ZEB2 protein translation. The lncRNAs acting with different enzymatic complexes are depicted with red squares.

LncRNAs engage in multiple cellular functions and regulate nuclear organization by acting as chromatin remodelers, thus affecting gene expression (Figure 2). Gene expression is also regulated by action at the RNA level and by sequestering of regulatory factors (transcription factors and catalytic proteins).

Many studies have demonstrated that lncRNAs are involved in several physiological and pathological phenomena, including proliferation, apoptosis, and DNA repair and response. A curated list of experimentally-supported associations between lncRNAs and diseases can be found at www.cuilab.cn/lncrnadisease.

In this review, attention is focused on the role of lncRNAs in adipogenesis and the regulation of adipose tissue function (Table 1). Further information on the lncRNA role in adipocyte biology and metabolism can be found in other recent review articles (Chen et al., 2018; Sun and Lin, 2019).

**Table 1.** Size and accession number of cited lncRNAs involved in adipogenesis.

	*Homo sapiens*	*Mus musculus*
Blnc1	759 nt URS0000DBDC4C_9606	965 nt URS0000D77FA2_10090
Lnc-BATE1		894 nt URS000075C5E3_10090
AK079912	N.D.	N.D.
LncBATE10		1808 nt URS000075F077_10090
MIAT	10,194 nt URS00007E4AF8_9606	9163 nt URS0000760956_10090
LINC01119*	1210 nt URS000075CEA4_9606	
LINC02202 *	2863 nt URS000075A437_9606	
*NONMMUG024827*	N.D	N.D.
H19*	2.362 nt URS0000812128_960	2286 nt URS0000767B73_10090
ASMER-1	N.D.	N.D.
ASMER-2	N.D.	N.D.
Plnc1	N.D.	N.D.
SRA	875 nt URS00001A8152_9606	829 nt URS00003EA2D2_10090
ADINR	2252 nt URS0000CCE086_9606	
HOTAIR*	2364 nt URS000075C808_9606	2222 nt URS000075BAE8_10090
PVT1*	1957 nt URS00008E3A67_9606	3319 nt URS000077AEFF_10090

*These lncRNAs have alternative transcripts. N.D. Not Detected in RNA database (https://rnacentral.org).

## Chromatin modification and remodeling

During the transcription phase, chromatin remodeling plays a crucial role in generating an open status (euchromatin), which is accessible to polymerases and transcriptional factors. Chromatin status can be modified by chromatin remodeling enzymes, which fall into two classes: ATP-dependent chromatin remodeling complexes and covalent histone modifiers (Figure 2 Panel A).

ATP-dependent chromatin remodeling complexes (SWI/SNF, NURD, CHRAC, etc.) disrupt histone-DNA interactions and increase nucleosome mobility along chromatin fibers. They may act either as co-activators or co-repressors, depending on the molecular context (i.e., interaction with activators or repressors, respectively) (Wu et al., 2009).

Histone covalent modifiers are enzymes that perform stable chemical modifications on histones. Changes in acetylation status of histones and/or methylation in specific amino acids (primarily lysine) can activate or deactivate the transcription process. Histone deacetylases (HDACs) and histone acetyltransferases (HATs), with their opposing activities, determine the pattern of histone acetylation. HDACs delete the acetyl groups from lysine residues on histone tails, which may play a critical role in gene repression. In contrast, HATs add an acetyl group on the same amino acid residues, promoting chromatin de-condensation and thus activating transcription (Di Bernardo et al., 2014).

Several histone methyltransferases and histone demethylases have been identified. These enzymes can influence transcription by acting as either co-activators or co-repressors, depending on the amino acid’s position. For example, SET1 methylates lysine in position 4 of histone H3 and activates transcription, while its corresponding demethylase LSD represses transcription. In contrast, SUV39H1/2 methylates lysine in position 9 of histone H3 and represses transcription, while its corresponding demethylase JMJD activates transcription (Thakore et al., 2016).

Other enzymes covalently modify nucleotides and contribute to chromatin remodeling. In the mammalian genome, one epigenetic mechanism that influences gene expression is associated with the specific DNA sequences called CpG islands. These are regions with a high frequency of CpG dinucleotides (Deaton and Bird, 2011). These sequences are principally found at over half of all promoter genes and can switch gene expression on or off, depending on cytosine methylation. Cytosine methylation occurs through the activity of DNA methyltransferases (DNMTs), while demethylation is performed by ten-eleven translocation (TET) family enzymes.

In this context, lncRNAs support gene regulation at the epigenetic level, acting with enzymes that modify histones and/or DNA, or with ATP-dependent chromatin remodeling complexes (Chalei et al., 2014; Grossi et al., 2020; Song et al., 2019).

In detail, some studies have demonstrated a functional interaction between DNMTs and lncRNAs (Dali, HOTAIR, Khps1) (Chalei et al., 2014; Di Ruscio et al., 2013; Postepska-Igielska et al., 2015; Zhao et al., 2016). For example, HOX transcript antisense intergenic RNA (HOTAIR) operates during epithelial-to-mesenchymal transition (EMT). This lncRNA is able to inhibit the transcription of E-cadherin switching of histone H3 lysine 27 acetylation to methylation at the gene promoter (Song et al., 2019).

## Transcriptional control

Different lncRNAs are involved in controlling the preliminary transcription stages (Figure 2 Panel B). These molecules play a role as cofactors that mediate interaction among activators and RNA Pol II preinitiation complex (PIC), which is assembled on TATA-dependent promoters (Ariel et al., 2020; Long et al., 2017). Some authors have suggested that lncRNAs acquire a specific R-loop structure, which contributes to transcription regulation. Specifically, antisense lncRNAs interact with duplex DNA strands of specific gene loci, forming a triple helix that can regulate sense mRNA transcription by recruiting cofactors (Yao et al., 2019).

LncRNAs can also regulate the elongation phase of the transcription process. LncRNAs, transcribed by RNA Pol II, interact with elongation factors, such as P-TEFb (Positive Transcription Elongation Factor b) (Price, 2000).

## Post-transcriptional control

The primary transcript of RNA polymerase II in eukaryotic cells undergoes several modifications during the transcriptional process, including 5’-capping, 3’-polyadenylation, and splicing. These events lead to mature mRNA, which is transported from the nucleus into the cytoplasm. LncRNAs are involved in several steps of mRNA maturation.

For example, MALAT1 modulates alternative splicing by interacting with splicing factors (SFs) such as SRSF1s, a conserved family of serine/arginine-rich (SR) proteins involved in RNA splicing regulation that is concentration- and phosphorylation-dependent (Figure 2 Panel C, top). MALAT1 localizes to interchromatin granule clusters (nuclear speckles) and regulates alternative splicing by modulating the distribution and levels of active SFs (Galganski et al., 2017; Tripathi et al., 2010).

LncRNAs like ZEB2/SIP1 NAT can bind splicing sites, suppressing them and resulting in intron retention. In the retained intron, the translation apparatus recognizes and binds an internal ribosome entry site (IRES), resulting in a more efficient ZEB2 protein translation (Bernardes de Jesus et al., 2018; Figure 2 Panel C, bottom). Some findings have exhibited the role of lncRNAs acting as NATs in increasing the stability of homologous mRNAs by forming a duplex, which masks the target binding site for miRNA and thereby increases the mRNA lifespan (Faghihi et al., 2010).

Other functions of lncRNAs have been described by Zhang and colleagues: they have shown a possible role in regulating protein stability through the block of the polyubiquitination that symbolizes the ‘kiss of death’ signal for protein turnover (Zhang et al., 2015).

Finally, lncRNAs can act as sponges, able to sequester miRNAs and thus avoid their binding with specific mRNA targets. In this way, mRNAs show greater stability and higher protein expression. An example is provided by the GUARDIN lncRNA. This molecule controls the expression of telomeric repeat binding factor 2 (TRF2), which is involved in the genomic integrity process under exogenous genotoxic stress. GUARDIN sequesters miR-23a, which has a specific target on TRF2 mRNA GUARDIN, and preserves TRF2 stability (Hu et al., 2018). LncRNAs can also bind and impair the activity of specific proteins in the cytoplasm (Lee et al., 2016).

## Specific expression of LncRNA in adipose tissues

**Figure 3.**
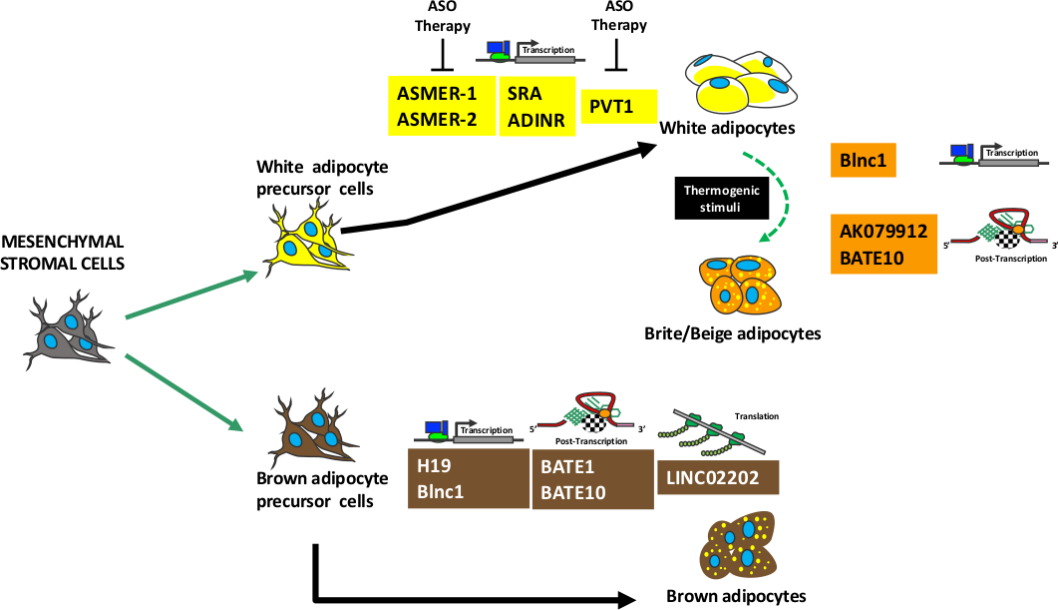
Summary of lncRNAs activity during adipogenesis. Mesenchymal stromal cells contain stem cells that can be committed to white or brown precursor cells able to differentiate in white or brown adipocytes, respectively. A specific subpopulation of white adipocytes can transdifferentiate in brite/beige adipocytes following thermogenic stimuli. The picture shows several lncRNAs involved in adipocyte maturation and/or in brite/beige transdifferentiation. For each lncRNA is indicated the molecular mechanism of action: regulation of transcription or post-transcription or translation. The lncRNAs that can be target of antisense oligonucleotide (ASO) therapy are depicted.

The lncRNAs have pleiotropic activities: a single lncRNA may regulate different biological functions depending on cellular context. The tissue specific expression of a given lncRNA it is not a pre-requisite for its peculiar activity, rather it depends on target molecules that are present in a given cell in a specific moment. Several lncRNAs, that are describe in the following paragraphs, are not exclusively expressed in adipose tissues, nevertheless they regulate adipose tissues’ specific functions. Figure 3 shows a summary lncRNAs activity during adipogenesis.

## LncRNAs as regulators of brown and brite/beige adipogenesis

Brite or beige adipocytes appear in WAT following thermogenic stimuli (Chen et al., 2016; Pfeifer and Hoffmann, 2015). They are dispersed within the WAT and have brown cell-like morphology, showing many small lipid droplets. Brite cells are rich in mitochondria and express the UCP1 protein involved in energy dissipation (Srivastava and Veech, 2019).

Recently, several studies have addressed the role of lncRNAs in the network governing adipogenesis and adipocyte biology. Some lncRNAs play a key role in these phenomena by interacting with different transcription factors, such as PGC-1alpha, EBF2, ZBTB7B, ZFP516, and PRDM16 (Cohen et al., 2014; Dempersmier et al., 2015; Harms et al., 2014; Li et al., 2017; Lin et al., 2005; Rajakumari et al., 2013; Seale et al., 2007).

Alvarez and colleagues used RNA-seq to identify 1500 lncRNAs expressed in brown, inguinal white, and epididymal white fat in mice. A subgroup of 127 lncRNAs was exclusively expressed in BAT; most of these targeted key regulators of adipogenesis, including PPAR-gamma, C/EBP-alpha, and C/EBP-beta (Alvarez-Dominguez et al., 2015). In this line, Zhao and colleagues were the first to identify a lncRNA that promotes brown and beige adipocyte differentiation. They named it brown fat lncRNA 1 (Blnc1) (Zhao et al., 2014).

Even though the molecular mechanism remains largely unknown, Blnc1 localizes in the nucleus and stimulates thermogenic gene expression via the Blnc1/hnRNP-U/EBF2 ribonucleoprotein complex. EBF2 (early B-cell factor 2) is a helix-loop-helix member of the EBF transcription factor family, which controls thermogenic brown and beige adipogenesis. Specifically, the interaction of Blnc1 with EBF2 promotes the increase of EBF2 transcriptional activity on the Ucp1 promoter. EBF2 controls PPAR-gamma binding activity on gene promoters to govern brown versus white adipocyte differentiation (Rajakumari et al., 2013; Shapira et al., 2017). Blnc1 is a specific target of the EBF2 gene and is necessary for its full transcriptional activity.

During adipogenic differentiation, a feedforward positive regulatory loop is generated: EBF2 is recruited onto the Blnc1 promoter and stimulates its transcription. Subsequently, a heterogeneous nuclear ribonucleoprotein (hnRNP-U) simulates a platform—used for the assembly of a molecular complex—that promotes the aggregation of transcribed Blnc1 and EBF2. This event induces the release of EBF2 from the Blnc1 promoter and the silencing of its transcription. The feedforward loop is relevant to the expression of genes regulating thermogenesis in brown adipogenesis.

Li and colleagues recently identified a transcriptional factor, named ZBTB7B (zinc finger and BTB domain-containing 7b), that promotes differentiation into brown and beige adipocytes and thermogenesis (Li et al., 2017; Mi et al., 2017; Zhao et al., 2018). ZBTB7B enrolls the hnRNP-U/Blnc1 complex to prompt expression of genes involved in nutrient oxidation and thermogenesis. As for EBF2, ZBTB7B is another key regulatory factor that interacts with lncRNA to form a regulatory pathway involved in adipogenesis and metabolism. Further investigation will clarify the role and type of epigenetic regulators engaged by Blnc1 (Mi et al., 2017).

Functional studies have highlighted that one more lncRNA is necessary for BAT induction, maintenance, and thermogenic activity. This RNA—lnc-BATE1—acts ‘in trans’ and is essential to brown adipogenesis; moreover, it also suppresses the white adipocyte selective gene program (Alvarez-Dominguez et al., 2015; Xiong et al., 2018). Interestingly, lnc-BATE1 regulates brown adipogenesis but is also involved (as with Blnc1) in the formation of a ribonucleoprotein complex that influences pre-mRNA processing and other cell activities related to transport and metabolism (Alvarez-Dominguez et al., 2015; Scheideler, 2019). How this ribonucleotide complex exerts its functions is not well understood and requires further investigation.

Xiong and colleagues have identified a brown adipocyte-enriched lncRNA they named AK079912. AK079912 expression is upregulated during brown adipocyte differentiation and following cold-stimulated browning of white adipocytes; accordingly, AK079912 silencing blocks the brown adipocyte differentiation. The action of this lncRNA has not been elucidated. Still, AK079912 contains an ORF that could be translated into a small peptide. This observation adds further complexity to the lncRNA mechanism of action, suggesting that a lncRNA may act either as noncoding RNA or as coding RNA. It is also possible that a region of an annotated lncRNA could give rise to a peptide and the rest of the molecule might function as a lncRNA (Xiong et al., 2018).

LncBATE10 is another lncRNA involved in brown adipogenesis (Bai et al., 2017). LncBATE10 expression drastically increases during browning induction. CELF1 (CUGBP Elav-like family member 1) is a possible protein partner for LncBATE10. CELF1 is an RNA-binding protein implicated in the regulation of such post-transcriptional events as pre-mRNA alternative splicing, mRNA translation, and stability. CELF1 can bind the 3’ UTR region of the PGC-1alpha mRNA, thus promoting its turnover (Gill and La Merrill, 2017). LncBAT10 can seize CELF1 and protect PGC-1alpha from degradation.

The transcription of lncBATE10 is under the control of the cAMP response element-binding protein (CREB) pathway (Bai et al., 2017). Indeed, the lncBATE10 promoter contains some CREB-responsive elements, and its transcription can be activated by CREB signal transduction. The adipocyte membrane exhibits the greatest expression of beta3-adrenergic receptors (β3AR), which are activated by adrenaline and noradrenaline during cold-induced browning. β3AR signaling activates adenylate cyclase, which produces intracellular cAMP to stimulate protein kinase A (PKA) (Ramseyer and Granneman, 2016; Guan et al., 1995; Tchivileva et al., 2009). Activated PKA modulates gene expression through phosphorylation of the CREB transcription factor, which in turn binds CRE (cAMP-response elements) sites in the promoter region of cAMP-responsive genes, such as lncBATE10 (Rockman et al., 2002).

Recently, Chen and colleagues have characterized three lncRNAs—MIAT, LINC01119, and LINC02202—that seem to play a key role during adipogenesis (Chen et al., 2019). In particular, MIAT might act as a sponge for hsa-miR-18a-5p miRNA by seizing it and preventing its action on the estrogen receptor 1 (ESR1) mRNA. LINC01119 might positively influence the transcription of protein tyrosine phosphatase receptor type B (PTPRB), which is involved in pathways that counteract adipose differentiation. In fact, PTPRB acts as a negative regulator of brown adipogenesis by suppressing the tyrosine phosphorylation of VEGFR2, and its expression decreases during BAT formation (Kim et al., 2019). By interacting with its cognate ligand (VEGF), the VEGFR2 receptor has a major role in brown adipose tissue development and maintenance (Bagchi et al., 2013). LINC02202 works as a competing endogenous RNA (ceRNA) for hsa-miR-136–5 p/hsa-miR-381–3 p miRNA in order to modulate PIK3R1 and FOXO1 gene expression. PIK3R1 is an essential element of the PI3K signaling pathway (PI3K/AKT), which is involved in adipogenesis through activation of specific transcription factors, such PPAR-gamma and C/EBP-alpha (Ambele et al., 2016).

A recent analysis compared adult and newborn BAT in mice. Several morphological and molecular differences were identified, including lncRNA expression (Liu et al., 2020). For example, neonatal BAT—which seems to be more physiologically plastic and active compared to adult BAT—expresses NONMMUG024827 lncRNA. This RNA is involved in positive regulation of adiponectin mRNA levels. Adiponectin is an adipocyte-specific protein, the reduction of which plays a central role in obesity-related diseases, insulin resistance/type 2 diabetes, glucose metabolism, and fat metabolism (Straub and Scherer, 2019). Adiponectin upregulation in neonatal BAT compared to adult tissue suggests that several glucose and lipid metabolism pathways may be more active.

Recent findings have led to the discovery of other lncRNAs that play a key role in BAT biology, such as the lncRNA H19 (Schmidt et al., 2018). This RNA modulates adipogenesis, oxidative metabolism, and mitochondrial respiration in brown—but not white—adipocytes. The lncRNA H19 binds the methylated DNA binding factor (MBD1 protein) and regulates the expression of some paternally-inherited genes (Igf2, Slc38a4 and MEST) that are mainly downregulated during BAT adipogenesis. Inverse correlation of H19 with BMI has been observed in humans, wherein its expression decreases in individuals with obesity (Schmidt et al., 2018).

## LncRNAs as regulators of white adipogenesis

In terms of BAT, lncRNAs take part in multiple gene expression networks that oversee WAT differentiation and its functions, both as energy reserve and as endocrine organ (Kershaw and Flier, 2004).

New investigations have shown a pivotal role for three lncRNAs—ASMER-1, ASMER-2, and Plnc1—during adipogenesis, which regulate the gene expression of key adipogenic transcription factors, such as PPAR-gamma (Gao et al., 2018; Zhu et al., 2019). ASMERs (adipocyte-specific metabolic related lncRNAs) and Plnc1 showed increased expression in adipose tissues from obese humans and mice, suggesting that an upregulation of their functions may be related to obesity. In particular, Plnc1 could increase PPAR-gamma2 expression by acting on its promoter and reducing the methylation status.

Gao and colleagues identified ASMER-1 and ASMER-2 in subcutaneous WAT obtained from patients with obesity and insulin resistance. They showed that in vitro silencing of ASMERs via antisense oligonucleotides suppressed adipogenesis, adiponectin production, and lipid mobilization (Gao et al., 2018).

Other attractive lncRNAs include SRA, ADINR, and HOTAIR, which are involved in fat accumulation (Xiao et al., 2015; Xu et al., 2010; Divoux et al., 2014).

Steroid receptor RNA activator (SRA) requires special attention. It was the first reported functional lncRNA present in fat cells, although its role in adipocyte differentiation appears undetermined. In vitro studies have suggested that SRA increases PPAR-gamma transcriptional activity in 3T3-L1 adipocytes (Xu et al., 2010). The SRA mechanism of action is not clear, since its gene locus is transcribed into three different isoforms, and only one these transcripts behaves like a ‘true’ lncRNA: the other two molecules display ORFs that could potentially be translated into two proteins (SRAPs) whose amino acid lengths are 224 and 236, respectively (Chooniedass-Kothari et al., 2004; Kawashima et al., 2003; McKay et al., 2014). Both SRA lncRNA and SRAPs could regulate the transcriptional activity of the estrogen receptor, which in turn can promote PPAR-gamma gene transcription (Coleman et al., 2004).

ADINR (adipogenic differentiation-induced noncoding RNA) was identified as a regulator of C/EBP-alpha, a critical transcriptional factor in adipogenesis (Xiao et al., 2015). In human undifferentiated MSCs, the C/EBP-alpha promoter is enriched in both activating and silencing the histone marks H3K4me3 and H3K27me3, respectively. During adipogenic differentiation, H3K4me3 increases at the expense of H3K27me3. It is assumed that ADINR orchestrates this complicated process by binding PA1, a member of the histone methylation complex MLL3/4 that contains H3K4me3 methylase and H3K27 demethylase. MLL3/4 activation via ADINR-PA1 increases K3K4me3 methylation, advantaging the transcription of the C/EBP-alpha locus.

HOTAIR (HOX transcript antisense intergenic RNA) is a lncRNA expressed in gluteal adipose depots without any sex distinction and with a possible role in the regulation of human subcutaneous pre-adipocyte differentiation (Divoux et al., 2014). Specifically, HOTAIR regulates the transcriptional silencing of genes in the HOXD locus, which is involved in adipocyte differentiation. HOTAIR creates a scaffold upon which two protein complexes cooperate to induce a heterochromatic status on the HOXD locus (Cowherd et al., 1997; Seifert et al., 2015).

Yang and colleagues have identified a lncRNA (UC001KFC.1) involved in glucose homeostasis (Yang et al., 2019). In obese subjects, white adipocytes show a decrease in UC001KFC.1 lncRNA expression. This lncRNA is a potential regulator of the PTEN protein, which is directly involved in modulating glucose uptake and enhancing insulin sensitivity. PTEN is a diphosphatase that converts PIP3 (phosphatidylinositol 3,4,5-trisphosphate) into PIP2 (phosphatidylinositol 4,5-trisphosphate). Research has shown that UC001KFC.1 reduction in obese subjects reflects a PTEN downregulation and a consequent PIP3 increase, which promotes glucose uptake via GLUT4 (Pan et al., 2017).

Leptin is secreted by white adipocytes and other cell types, and it plays a key role in regulating the body’s intake and energy consumption (Caron et al., 2018). Findings have shown that leptin regulates the Hedgehog pathway, playing a critical role in adipogenesis and in white adipocyte browning (Wang et al., 2019a). Stringent control of leptin expression is fundamental to physiological regulation of white adipocyte biology and energy consumption. In this context, a genome-wide meta-analysis has enabled identification of new gene loci that influence circulating leptin levels (Kilpeläinen et al., 2016). Another finding has shown that leptin expression is controlled by cis elements and trans factors interacting with the leptin proximal promoter, together with a lncRNA (lncOb). Obese mice lacking this lncRNA showed increased fat mass with reduced circulating levels of leptin; these mice lose weight after leptin supplementation, whereas control mice do not (Dallner et al., 2019).

The lncRNA PVT1 could be a potential target for obesity treatment, since its expression is positively associated with full differentiation of 3T3-L1 preadipocytes. Moreover, PVT1 expression in WAT of obese mice is higher than that in WAT of healthy mice. PVT1 appears to be involved in lipid accumulation in white adipocytes by upregulating the expression of peroxisome proliferator activated receptor gamma, CCAAT/enhancer-binding protein α, and adipocyte protein 2 (Zhang et al., 2020).

An interesting study has enabled the identification of Mist, a lncRNA expressed in peritoneal macrophages and adipose tissue macrophages. Mist downregulation was associated with the expression of pro-inflammatory factors, as it occurs in obesity. Mist regulates the expression of the PARP1 gene that is involved in DNA repair mechanisms. Disruption of Mist-PARP1 interaction increases PARP1 recruitment onto the promoters of inflammatory genes, resulting in increased gene expression (Stapleton et al., 2020).

## Role of lncRNAs in adipose tissue biology: CAVEATS

The ncRNAs display an amazing variety of sizes, forms and functions. The classification in lncRNAs and sncRNAs is somewhat arbitrary and may hamper the discovery of their mechanism of actions. Indeed, the functions of many sncRNAs are well defined, while further studies are needed to completely dissect the molecular mechanisms of lncRNAs. In this context a further complexity derives from investigations showing that some lncRNAs may be translated and produce short peptides.

All together these caveats represent a hurdle for a complete knowledge of lncRNA role in adipose tissue development and activity.

## Conclusion

Adipogenesis appears to be a process that is characterized by an intricate network in which many transcription factors and lncRNA are involved as regulators of gene expression.

In this review, some examples have been provided of a new class of RNA molecules that participate in a wide range of cellular functions during white and brown/beige adipogenic development and activity. The vast majority of clinical drugs target proteins (Wishart, 2006); however, these often have side effects, since they can also interact with non-target proteins. Drugs that target nucleic acids are an emerging class of therapeutics for treating unmet medical needs, since they may present fewer side effects compared with existing therapies (Arun et al., 2018).

Currently, certain important drugs target nucleic acids in the areas of antibacterial and anticancer therapy. Targeting lnCRNAs for therapy relies on different approaches. For example, deregulated high lncRNA levels may be lowered with antisense oligonucleotides (ASO) that block lncRNA activity and induce their degradation. Alternatively, lncRNA function may be blocked by small molecules that mask the binding site of interacting proteins, or by antisense oligonucleotides that bind to the lncRNAs and inhibit their protein binding capacity (Sánchez and Huarte, 2013).

In detail, the lncRNAs regulating brown adipogenesis and BAT functions cannot be target of antisense therapy, since their silencing with ASO will impair BAT activities. On the contrary, there are many lncRNAs (ASMERs, Plnc1, ADINR, PVT1) that positively regulate white adipogenesis and are upregulated in obese individuals. These molecules are suitable ASO targets. Preliminary in vitro studies (Gao et al., 2018) showed that ASO approach could be a valuable tool for treatment of obesity. The feasibility of ASO therapy for targeting lncRNAs has been demonstrated in some pre-clinical models. Antisense phosphorothiote oligonucleotides were used to target lncRNAs involved in Angelmann syndrome and lung cancer in mice (Matsui and Corey, 2017).

The study of lncRNAs in adipogenesis and related diseases, along with the therapeutic approaches described above, may pave the way to developing new strategies for fighting obesity and related metabolic diseases.
